# Impact of Rocky Desertification Control on Soil Bacterial Community in Karst Graben Basin, Southwestern China

**DOI:** 10.3389/fmicb.2021.636405

**Published:** 2021-03-10

**Authors:** Qiang Li, Ang Song, Hui Yang, Werner E. G. Müller

**Affiliations:** ^1^Key Laboratory of Karst Dynamics, MNR and GZAR, Institute of Karst Geology, Chinese Academy of Geological Sciences, Guilin, China; ^2^International Research Center on Karst Under the Auspices of UNESCO, Guilin, China; ^3^ERC Advanced Investigator Grant Research Group, Institute for Physiological Chemistry, University Medical Center of the Johannes Gutenberg University Mainz, Mainz, Germany

**Keywords:** ecological type, 16S amplicon sequencing, co-occurrence network, karst rocky desertification control, karst graben basin

## Abstract

Microorganisms play critical roles in belowground ecosystems, and karst rocky desertification (KRD) control affects edaphic properties and vegetation coverage. However, the relationship between KRD control and soil bacterial communities remains unclear. 16S rRNA gene next-generation sequencing was used to investigate soil bacterial community structure, composition, diversity, and co-occurrence network from five ecological types in KRD control area. Moreover, soil physical-chemical properties and soil stoichiometry characteristics of carbon, nitrogen and phosphorus were analyzed. Soil N and P co-limitation decreased in the contribution of the promotion of KRD control on edaphic properties. Though soil bacterial communities appeared strongly associated with soil pH, soil calcium, soil phosphorus and plant richness, the key factor to determine their compositions was the latter via changed edaphic properties. The co-occurrence network analysis indicated that soil bacterial network complexity in natural ecosystem was higher than that in additional management ecosystem. *Candidatus Udaeobacter*, *Chthoniobacterales*, and *Pedosphaeraceae* were recognized as the key taxa maintaining karst soil ecosystems in KRD control area. Our results indicate that natural recovery is the suitable way for restoration and rehabilitation of degraded ecosystems, and thus contribute to the ongoing endeavor to appraise the interactions among soil-plant ecological networks.

## Highlights

- Returning farmland to forests in karst graben basin enhances soil prosperity.
- Plant species richness shapes soil bacterial communities via the changed edaphic properties.
- Soil *Candidatus Udaeobacter*, *Chthoniobacterales*, and *Pedosphaeraceae* play key roles in biogeochemical cycles of organic materials.
- Soil bacterial network complexity in natural ecosystem is higher than that in additional management ecosystem.
- Natural recovery is the suitable way for restoration and rehabilitation of degraded ecosystems.

## Introduction

Karst environment is fragile in southwest China due to the particularities of soluble carbonate rock, rich calcium, soil scarcity and water leakage, etc (Yuan, 2001). Under the pressure of 1.7 million people living there, forest, especially at the slope >25° area, was destroyed for agriculture; consequently, an ecological disaster, named karst rocky desertification (KRD), relating to deforestation and soil loss, was formed (Yuan, 1997; Jiang et al., 2014). KRD control project (returning farmland to forests) was applied to recover the disturbed karst ecosystem in southwestern China, which may affect soil physical, chemical and biological properties (Jiang et al., 2014; Li et al., 2014; Chang et al., 2018; Bai et al., 2019) and vegetation coverage (Jiang et al., 2014; Tong et al., 2018).

Microorganisms are important components of soils (Beveridge, 1989), which play major roles in maintaining biogeochemical cycling, complexity and stability of belowground ecosystems (Delgado-Baquerizo et al., 2018, 2019; Wagg et al., 2019). Predictive understanding of soil bacterial communities is very important to forecast the ecological consequences caused by environmental changes (Delgado-Baquerizo et al., 2018; Liang et al., 2020). Besides, the advent of high-throughput sequencing technologies provide a rapid and low-cost tool to understand microbial ecology, such as microbial community structures, diversities, interactions and co-occurrence patterns (Loman et al., 2012; Ma et al., 2016; Xue et al., 2017, 2020; Schmidt et al., 2019; Wagg et al., 2019).

Previous researches also have shown that soil bacterial communities can be shaped by edaphic properties such as pH, Ca (Paul, 2007; Xue et al., 2017; Delgado-Baquerizo et al., 2018, 2019; Li et al., 2021) and plant factors such as species structure and diversity (Marschner et al., 2001; Van der Heijden et al., 2008; Lamb et al., 2011; Lange et al., 2014; Dassen et al., 2017; Ma et al., 2019; Zhong et al., 2020). However, the characterizing changes in soil bacterial community structure and diversity in response to KRD control remains still unclear. This is unsurprising given that soil, the continuous spatio-temporal heterogeneity with high spatial variability, harbors vast soil microorganisms (Yuan, 1997; Harris, 2009; Jiang et al., 2014; Delgado-Baquerizo et al., 2018, 2019), and natural and artificial ecosystems after returning farmland to forests are different (Swenson et al., 2000). Put simply, some bacteria are highly abundant under particular environmental conditions (Ma et al., 2016; Delgado-Baquerizo et al., 2018). For these reasons, we lack systematic understanding of the interactions among KRD control and soil bacterial communities in a given karst area.

To reduce the knowledge gap, the “space-for-time substitution” approach (Pickett, 1989) and high-throughput sequencing of the 16S rRNA gene were applied to explore the characterizing changes of soil bacterial community structure and diversity, and their responses to returning farmland to forests from KRD control area in Hani-Yi Autonomous Prefecture of Honghe, Yunnan Province, China. Though “space-for-time substitution” approach has shortcomings, it is still recognized as the only way of determining long-term ecological dynamics (Wogan and Wang, 2018). Then, the starting point of this study is the question whether the soil bacterial community difference exists among different ecology types along the successional gradients in KRD control area. Another question is: if the difference exists, do you provide any evidence to determine the soil eco-stability with ecological type? Finally, we want to reveal the determining factor(s) shaping soil bacterial communities in our study.

## Materials and Methods

### Study Area

Hani-Yi Autonomous Prefecture of Honghe (101°47´–104°16´E, 22°26´–24°45 N) is located at the juncture of the Tethys and Pacific tectonic domain, resulting in the formation of sedimentary basins of graben type over the Yunnan Plateau (Qin et al., 1996; Duan et al., 2005; Li et al., 2021). Nearly 55% of this autonomous prefecture of 33430 km^2^ is karstic. The KRD area covered 2345.08 km^2^ in 2016 (Zhao et al., 2019). Karst soil layers are usually 20–40 cm thick. About 80% of annual rainfall (2026.5 mm) concentrates in the rainy season onset of May but end of the October (Duan et al., 2005), which falls within the subtropics with two distinctive seasons (dry and wet seasons). The average annual temperature is 16.3°C.

### Bulk Soil Sampling

**FIGURE 1:**
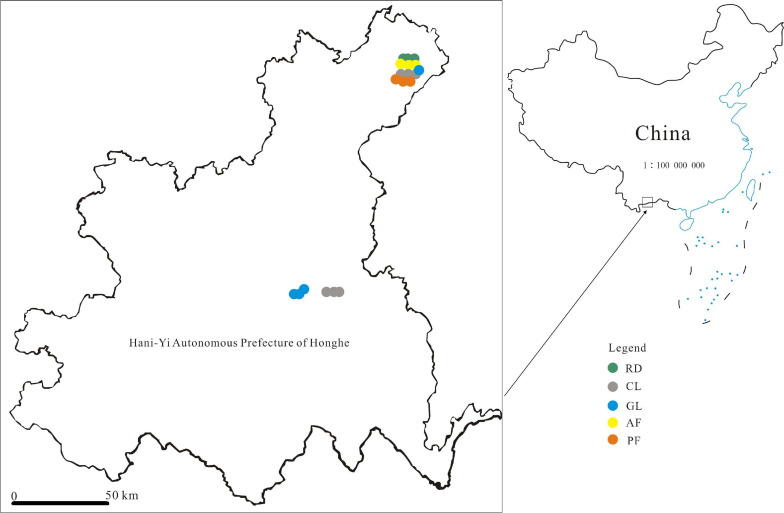
Map showing localization of the bulk soil samples in Hani-Yi Autonomous Prefecture of Honghe, China. Figure 1 was generated by QGIS that is a free and open source geographic information system (https://www.qgis.org/en/site/).

Nineteen plots with the size of approximately 10 × 10 m^2^ were randomly selected for bulk calcareous soil sampling in Oct, 2016. In the total 19 plots (Figure 1), there are six plots from corn land (CL) with *Zea mays*, four plots from grassland (GL) covered mainly by *Arundinella setosa*, three plots from natural restoration forest with *Alnus ferdinandi-coburgii* Schneid (AF), three plots from artificial restoration forest with *Pinus yunnanensis* forest (PF) and three plots from rock covered soil without disturbance (RD) covered mainly by *Bauhinia brachycarpa*, *Cymbopogon distan*s and *Cyclobalanopsis glaucoides* Schottky. Each ecological plot has been used for more than 10 years. 264:75:13.5 kg N:P_2_O_5_:K_2_O/hm^2^ and 15,000 kg organic fertilizer/hm^2^ were applied on the CL soil every year, and GL, AF as well as PF recovery from CL were under natural restoration without human intervention. Considering the heterogeneity of sampling plots, plant biodiversity was investigated in term of Gleason index (the average plant Gleason index in CL, GL, AF, PF, and RD were 2.8, 7.7, 7.3, 5.4, and 9.6, respectively). When bulk soils were sampling, the sampling plots were uniformly distributed along an S-type, and every three diagonal sampling cores with 5 cm diameter and 10 cm depth in each plot were combined to obtain a composite soil sample. After stones, plant as well as root debris removed, the 19 soil samples in total were used for downstream analysis.

### Analysis of Bulk Soil Properties

Soil pH, soil organic carbon (SOC), total nitrogen (TN) and total phosphorus (TP) were analyzed by using the air-dried and sieved (0.2 mm) soil samples (Li et al., 2015a). The exchangeable bases calcium (E-Ca) and magnesium (E-Mg) were extracted using an ion-exchange resin (McKinley et al., 2007). The soil properties are summarized in Table 1.

**TABLE 1:** Mean soil properties in five ecological types.

Type	pH	SOC	TN	TP	E-Ca	E-Mg	C:N	C:P
				
		g/kg	g/kg	mg/g	mg/k	mg/g		
CL	6.65 ± 0.16a	27.13 ± 7.69b	0.88 ± 0.15c	1.43 ± 0.51b	4.60 ± 0.55b	5.93 ± 0.40*ab*	30.47 ± 1.64a	22.21 ± 4.93b
GL	6.73 ± 0.13a	24.93 ± 5.66b	0.83 ± 0.21c	0.89 ± 0.16c	4.15 ± 1.21*bc*	4.36 ± 1.26b	30.18 ± 1.01a	28.04 ± 1.91*ab*
PF	6.71 ± 0.14a	32.42 ± 2.48b	1.21 ± 0.04b	2.04 ± 0.13a	6.03 ± 0.67a	5.14 ± 0.50b	26.71 ± 0.80*ab*	16.00 ± 1.28b
AF	6.24 ± 0.09b	33.13 ± 1.82b	1.30 ± 0.04b	1.27 ± 0.03*bc*	2.99 ± 0.12c	5.26 ± 0.14b	25.44 ± 0.39b	26.12 ± 1.04*ab*
RD	6.68 ± 0.08a	43.67 ± 4.18a	1.69 ± 0.15a	1.15 ± 0.07*bc*	5.13 ± 0.36*ab*	6.77 ± 0.39a	25.85 ± 0.16b	37.94 ± 1.49a

*Values are the mean of analytical replicates for each sample ± standard deviations. Statistical pairwise multiple comparisons of data homogeneity were carried out by the LSD test: means with the same letter in the same column are not significantly different at *P* < 0.05.*

### High-Throughput Sequencing and Bioinformatic Analysis

Soil DNA was extracted from 0.25 g fresh soil by using PowerSoil DNA Isolation Kit (Mobio Laboratories, Inc., Carlsbad, CA, United States) in strict accordance with the manufacturer’s instructions.

PCR primers 515F (5´-GTGCCAGCMGCCGCGGTAA-3´) and 907R (5´-CCGTCAATTCMTTTRAG TTT-3´) with a 12-bp barcode sequence at the 3´ end were used for amplification of the V4-V5 region of 16S rRNA genes (Tamaki et al., 2011). The PCR amplification (60 μl) were done in 6 μl 10× Ex Taq buffer, 6 μl dNTP mixture, 0.6 μl BSA, 0.3 μl Ex Taq, 1.2 μl of each primer, 1 μl DNA template, 43.7 μl sterile deionized water (Guangzhou MAGIENE Biotech Co., Ltd., China). PCR conditions were 5 min at 94°C followed by 31 cycles of 30 s at 94°C, 30 s at 52°C and 45 s at 72°C followed by a final extension for 10 min at 72°C. After the PCR amplification, samples were held at 16°C for 10 min and then were analyzed on 1% agarose gel. High quality PCR products were purified by using the TIANquick Maxi Purification Kit [TIANGEN Biotech (Beijing) Co., Ltd., China] and sequencing was performed on the Illumina HiSeq 2500 platform (Illumina Inc., San Diego, CA, United States) to generate 250 bp paired–end reads in the company of MAGIENE (Guangzhou, China). The achieved raw sequence reads were submitted to the NCBI Sequence Read Archive under the accession numbers PRJNA471031 and PRJNA471162.

Raw sequencing reads were processed by QIIME version 1.9.1. Sequences with low quality (length <300 bp or average base quality score <20) were removed. Chimeric reads were identified and excluded through the Usearch (version 5.2.236). Moreover, reads which were not classified into bacteria and annotated as chloroplast and mitochondria were discarded. Then, 1481930 raw reads, with a minimum sequencing depth of 60317 reads per sample, resulted in 1426986 high–quality sequences. The clean reads were grouped into operational taxonomic units (OTUs) based on a genetic similarity of 97% by using Usearch version 5.2.236. To ensure that well–characterized strains were included in the phylogenetic analysis, the 16S rRNA gene sequences of type strains from each of the target taxa were extracted from the Silva version 132 16S rRNA database. In this study, soil bacterial communities are focused; consequently the detailed data of 16S rRNA gene sequencing except for archaea are summarized in Supplementary Table 1.

### Data Analysis

In our study, the C:N and C:P ratios were calculated based on the ratios between concentrations in each of the individual soil samples (SOC/TN and SOC/TP, all in g/kg). Alpha diversity measures (Chao 1, Simpson, Shannon, observed OTUs, Goods coverage and Fisher index) and soil properties were used for significance analysis followed by one–way–analysis of variance with least–significant difference (LSD) at 0.05 level using SPSS version 19.0 software (IBM Corp., Armonk, NY, United States) to explore their distribution patterns in different ecological types. To detect the strength of edaphic drivers upon bacterial communities’ structure and OTU associations with ecological types, a redundancy analysis (RDA) was made by CANOCO for Windows 4.5 (Ithaca, NY, United States). The R environment^1^ with version 3.6.3 was also performed for statistical computing. Principal coordinate analysis (PCoA) plots were drawn to assess the similarity of soil bacterial community structures based on weighted Unifrac and Bray–Curtis distances by applying the vegan package in R. Interactions with *P*-values < 0.01 and a false discovery rate <0.05 among the most abundant OTUs and between these OTUs and edaphic variables were kept to generate correlation networks by using Gephi version 0.9.2 (Barberán et al., 2012). Heat map illustrating the correlations between edaphic variables and OTUs with *P*-values < 0.05, and the relative frequencies of the most abundant OTUs (>0.5%) was created by the pheatmap R package. The co-occurrence of OTUs in soil bacterial communities across five ecological types was also analyzed. To reduce network complexity and facilitate the identification of the core soil bacterial communities, OTUs with more than five sequences were selected for further analysis (Barberán et al., 2012). A valid co-occurrence event to be a robust correlation was considered by using Gephi, when the Spearman’s correlation coefficient (*r*) was >0.6 and statistically significant (*P*) was <0.01 (Junker and Schreiber, 2011; Barberán et al., 2012; Ma et al., 2016). Partial Least Squares Path Modeling (PLS-PM; Tenenhaus et al., 2005; Zhong et al., 2020) was used to explore the relationships among soil bacterial community compositions (including main phyla with the relative abundance higher than 0.1%) and alpha diversities, soil properties (pH, SOC, TN, TP, E-Ca and E-Mg), and plant richness factors (plant Gleason index) by using the plspm package, boot package and vegan package in R. Their correlations were evaluated by using PASSaGE 2 based on Partial Mantel (PM) test, which could eliminate the collinearity of variables. Variation partitioning analysis (VPA) was performed using the Vegan package in R to quantitatively evaluate the contribution of soil properties and plant richness factors to the variation of soil bacterial community compositions and alpha diversities. Analysis of similarity (ANOSIM) tests was conducted with the vegan R package to determine the statistical differences in five ecological types by permutation of group membership with 999 replicates, where the test statistic R, which measures the strength of the results, ranges from -1 to 1 (*R* = 1 signifies differences between groups, while *R* = 0 signifies that the groups are identical; Anderson and Walsh, 2013).

## Results

### Bulk Soil Physicochemical Characteristics

Soil SOC, TN, and E-Mg contents increased from GL to PF, AF, and RD after returning farmland to forests, which exhibited significant differences between RD and other ecological types. Soil pH values showed a high spatial heterogeneity, though the significant difference in soil pH values was observed between RD and other ecological types. High spatial heterogeneity in soil TP and E-Ca contents, and soil C:P ratios were also observed among five ecological types. In contrast, soil C:N ratios were clearly decreasing along the successional gradients from CL to RD. Interestingly, soil pH values, soil SOC, TN, E-Ca, and E-Mg contents, and soil C:P ratios in CL were lower than those in RD, though soil TP contents and C:N ratios in CL were higher than those in RD.

### Soil Bacterial Community Structure and Diversity Under the Influence of Ecological Types

**FIGURE 2:**
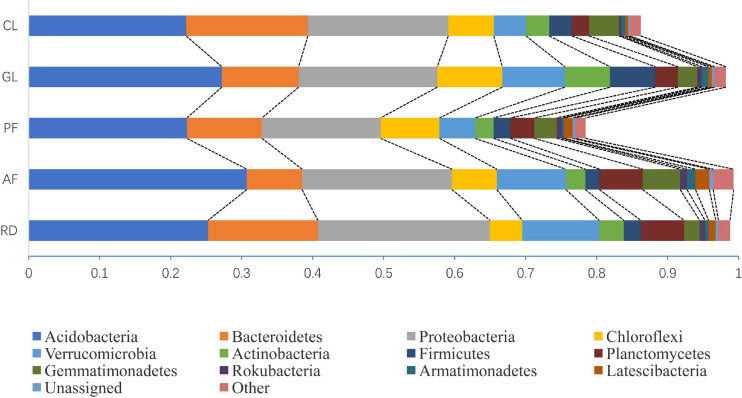
Comparison of the quantitative contribution of the sequences affiliated with different bacterial phyla to the total number of sequences from five ecological types. Sequences not classified to any known phylum are included as unassigned bacteria. In each ecological type, bacterial phyla with a largest relative frequency of less than 0.1% are included as others.

Of all the reads, we found that >77.92% of the 16S sequences were assigned to bacteria, where >69.79 % of the 16S sequences were assigned to 12 bacterial phyla (every sample’s relative frequency >0.1%), with the remainder being un-assigned to a known bacterial phylum. The dominate phyla Acidobacteria, Bacteroidetes, Proteobacteria, Chloroflexi, Verrucomicrobia, Actinobacteria, Firmicutes, Planctomycetes, Gemmatimonadetes, Rokubacteria, Armatimonadetes, and Latescibacteria had the different mean relative frequency across five ecological types (Figure 2).

**FIGURE 3:**
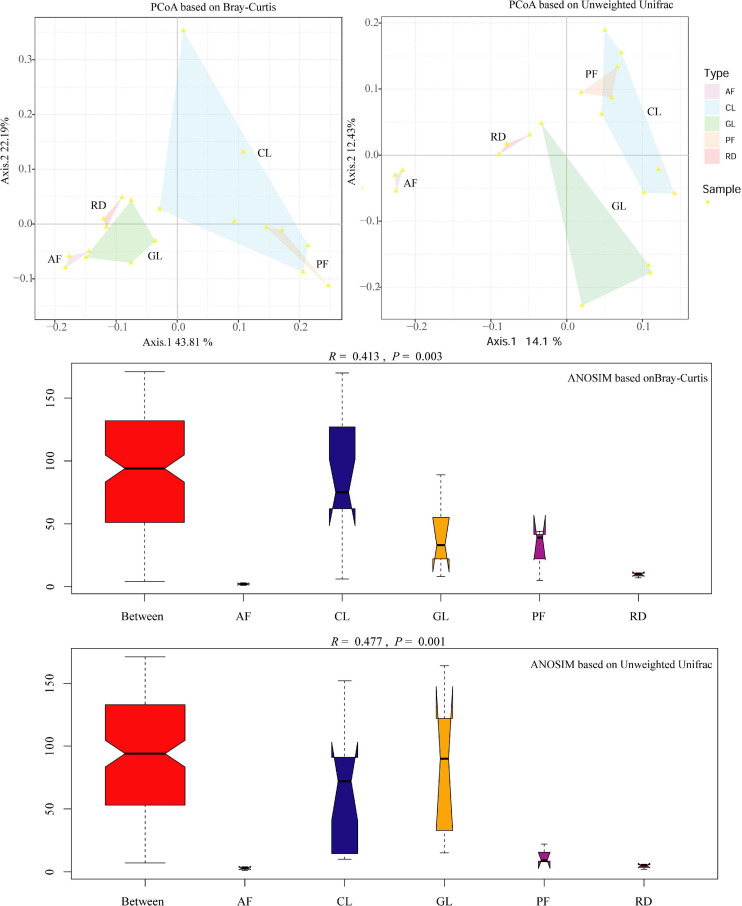
PCoA plots and ANOSIM analysis based on unweighted Unifrac and Bray-Curtis distances representing the soil bacterial community similarity/dissimilarity. Ordinate–the rank of the distance between samples; Abscissa–Between is the result between the five ecological types, and the other five are the results within their groups, respectively.

In general, soil bacterial alpha diversity indices increased from CL to GL, AF/PF and RD (Table 2). The difference of soil bacterial beta diversity was visualized by the PCoA tool with an unweighted UniFrac and Bray–Curtis distance matrix, though PF and CL had some overlaps. Moreover, ANOSIM revealed that KRD control exerted a strong influence on the soil bacteria communities with significant statistical differences (Bray-Curtis—*R* = 0.413, *P* = 0.003, and unweighted UniFrac—*R* = 0.477, *P* = 0.001), as seen in Figure 3. The two relations in five ecological types based on ANOSIM have shown that AF/CL, AF/GL and CL/RD had the significant statistical differences (*P* < 0.001) (Supplementary Figure 1).

**TABLE 2:** Mean alpha diversity in five ecological types.

Type	Chao 1	Shannon	Simpson	Observed OTUs	Goods coverage (%)	Fisher index
CL	1987 ± 55b	7.06 ± 0.17b	0.96 ± 0.01b	1390 ± 33b	97.23 ± 0.09*ab*	344.75 ± 0.83b
GL	1906 ± 121b	7.28 ± 0.23b	0.97 ± 0.01*ab*	1403 ± 79b	97.36 ± 0.17a	349.56 ± 26.37b
PF	2165 ± 42*ab*	7.15 ± 0.29b	0.94 ± 0.02b	1503 ± 29*ab*	96.99 ± 0.04b	382.08 ± 9.61*ab*
AF	2252 ± 71a	8.43 ± 0.05a	0.99 ± 0.00a	1683 ± 55a	96.98 ± 0.10b	444.93 ± 19.72a
RD	2231 ± 79a	8.32 ± 0.19a	0.99 ± 0.00a	1664 ± 69a	97.04 ± 0.09*ab*	438.34 ± 24.48a

*Values are the mean of analytical replicates for each sample ± standard deviations. Statistical pairwise multiple comparisons of data homogeneity were carried out by the LSD test: means with the same letter in the same column are not significantly different at *P* < 0.05.*

### Distribution of Soil Bacterial Community Compositions in Five Ecological Types

**FIGURE 4:**
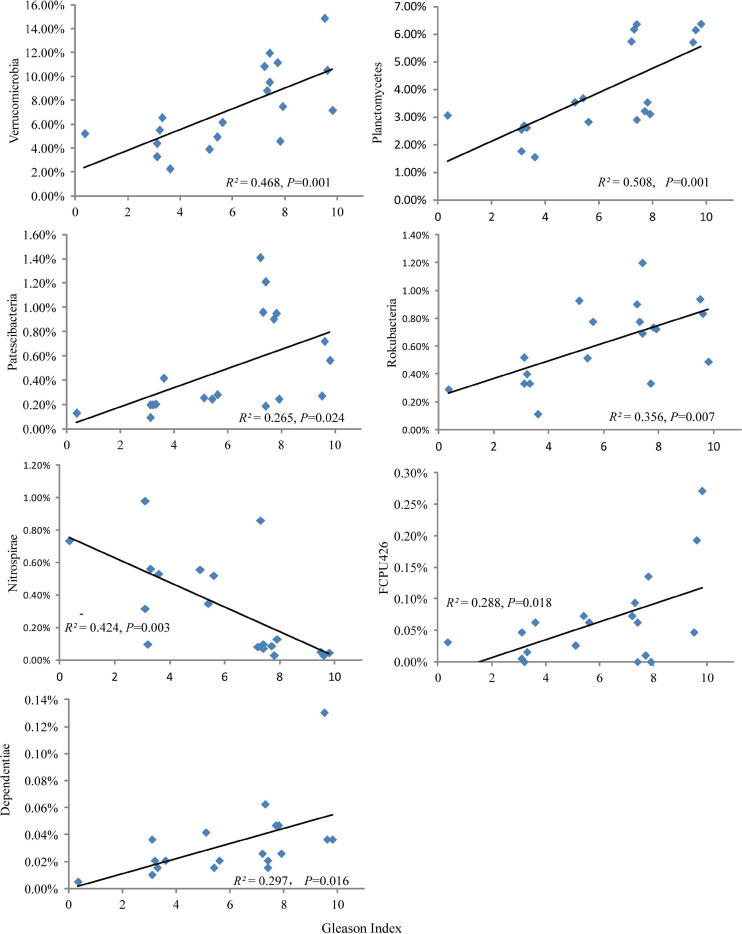
Changes in the relative abundances of soil bacterial phyla with plant Gleason index.

**FIGURE 5:**
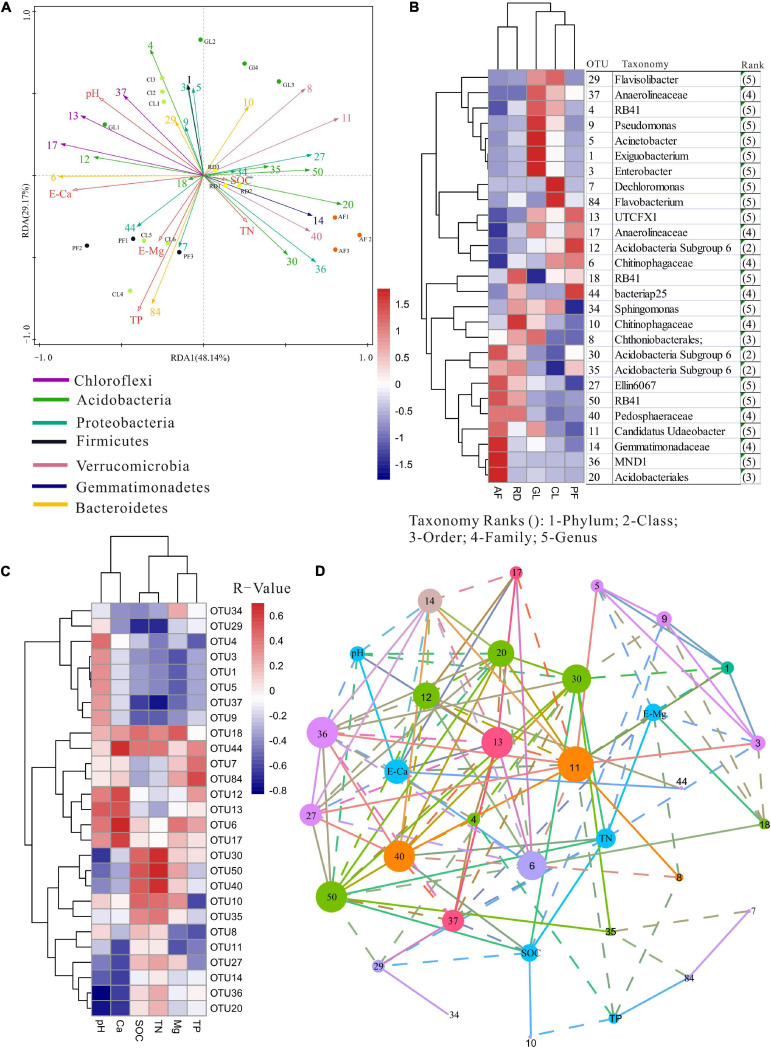
**(A)** RDA plots showing the relationship between samples (color corresponds to ecological types), 27 top OTUs (color corresponds to taxonomic affiliation) and edaphic variables (red arrows). **(B)** Heat map illustrating the relative frequency of the 27 most abundant OTUs. **(C)** Heat map representing clustering between edaphic variables and the 27 top OTUs, which had at least one *R*-value > 0.5 or <–0.5. **(D)** Correlation network of significant positive and negative correlations among OTUs (node color corresponds to taxonomic affiliation) and between OTUs and edaphic variables. Node size is proportional to the OTU abundance.

The relative abundances of Verrucomicrobia, Planctomycetes, Patescibacteria, Rokubacteria, FCPU426, and Dependentiae increased significantly with plant Gleason index, while the relative abundances of Nitrospirae decreased significantly with plant Gleason index (Figure 4). Moreover, the heat map clearly suggested that ecological types had a greater effect on most frequent OTU occurrences (Figure 5B). However, only *Candidatus Udaeobacter* (OTU11), *Chthoniobacterales* (OTU8) and *Pedosphaeraceae* (OTU40) were detected as the most frequent bacteria taxa in the dominate phylum Verrucomicrobia (Figure 5B) and increased significantly with plant Gleason index (Supplementary Figure 2).

### Relationships of Soil Bacterial Communities With Edaphic Properties in Five Ecological Types

Redundancy analysis revealed that the distribution of 27 top OTUs was strongly affected by ecological types (Figure 5A). Permutation test analysis revealed that the 27 top OTUs distribution across all samples could be mainly explained by the RDA1 axis (*F* = 4.4, *P* = 0.002), significantly correlating with E-Ca (*F* = 5.5, *P* = 0.002), TP (*F* = 4.3, *P* = 0.006), pH (*F* = 3.9, *P* = 0.004) and plant Gleason index (*F* = 2.4, *P* = 0.048). Moreover, the edaphic variables explained 73.5% of the soil bacterial variance, with axis 1 explaining 33.81% of the variance and axis 2 explaining another 21.09%. A Pearson correlation-based heat map was drawn to illustrate associations between the most abundant OTUs and edaphic variables (Figure 5C). Network analysis showed associations between co-occurring OTUs with edaphic variables (Figure 5D), e.g., the most frequent Verrucomicrobia OTUs (8 and 11) negatively related to TP and E-Mg, and OTU 40 positively related with TN and SOC, as also seen in Figures 5A,C.

**FIGURE 6:**
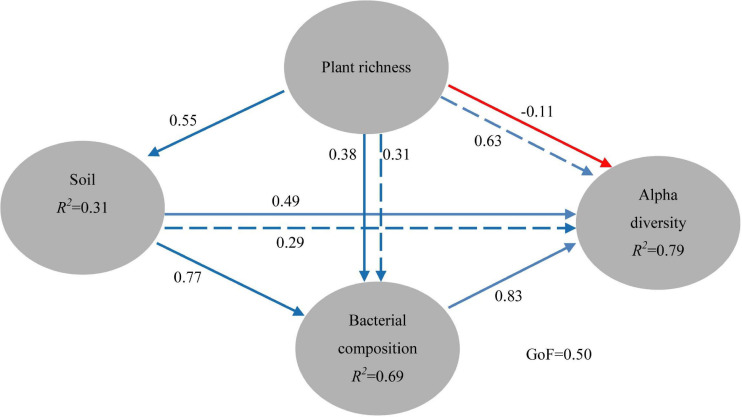
Directed graph of the PLS-PM of plant richness effects on soil properties (pH, SOC, TN, TP, E-Ca, and E-Mg), and soil bacterial community composition (main phyla with the relative abundance higher than 0.1%) and alpha diversity (Chao 1, Simpson and Shannon, observed OTUs, Goods coverage, Fisher index). The path coefficients and the explained variability (*R*^2^) in our study were calculated after 999 bootstraps. Blue solid arrows indicate positive direct effects, red solid arrows indicate negative direct effects, blue dashed arrows indicate positive indirect effects. Models with different structures were assessed using the Goodness of Fit (GoF) statistic, a measure of the overall prediction performance. For the PLS-PM represented here, the GoF was 0.50.

To better explore the key drivers shaping soil bacterial communities in five ecological types, a variety of statistical methods was used in our study. The PM test showed that plant richness (that is, Gleason index) had the significant (*r* = 0.314, *P* = 0.002) effects on soil properties (pH, SOC, TN, E-Ca, E-Mg, and TP). Plant richness or soil properties had no significant (*P* > 0.05) effects on soil bacterial community composition (Table 3). Though plant richness had no significant (*P* > 0.05) effects on soil bacterial alpha diversity, soil properties had the significant (*P* < 0.01) effects on soil bacterial alpha diversity (Table 3). C: P ratios also have the significant influence on soil bacterial community composition (*r* = 0.29, *P* = 0.010). Besides, the Partial Least Squares (PLS) path model was applied in our study to integrate the complex interrelationships among edaphic factors and soil bacterial communities (Figure 6). The PLS model was represented here with the GoF 0.50. According to the PLS-PM, plant richness exerted significant direct effects on soil properties (*r* = 0.55, *P* = 0.017), which exerted significant direct effects on soil bacterial community composition (*r* = 0.77, *P* = 0.005) and exerted significant effects on soil bacterial alpha diversity (*r* = 0.78, *P* = 0.023). Interestingly, the changes of bacterial community composition had the significant direct effect on soil bacterial alpha diversity (*r* = 0.83, *P* = 0.034). Moreover, the variance partitioning showed that plant richness can explain 5.6% and 34.0% of the variation about bacterial community composition and alpha diversity, soil properties can explain 34.0% and 35.6% of the variation about bacterial community composition and alpha diversity, and the variations of bacterial community composition and alpha diversity are mainly explained by soil properties (34.1%) and plant richness (34.0%) (Supplementary Figure 3).

**TABLE 3:** Influence of plant richness and soil properties on bacterial community composition and alpha diversity by partial Mantel test.

Effect of		Plant richness^a^		Soil		C:N ratios	C:P ratios
Controlling for			Soil^b^		Plant richness		
Bacterial community composition^c^	Correlation	0.156	0.116	0.148	0.105	0.033	0.291
	*P*-value	0.217	0.301	0.328	0.453	0.823	0.010
Alpha diversity^d^	Correlation	0.053	−0.052	0.319	0.319	0.026	0.015
	*P-*value	0.617	0.607	0.009	0.006	0.829	0.975

*^*a*^Plant richness includes plant Gleason index. ^*b*^Soil properties (pH, SOC, TN, TP, E-Ca, and E-Mg). ^*c*^Soil bacterial community composition (main phyla with the relative abundance higher than 0.1%). ^*d*^Alpha diversity (Chao 1, Simpson and Shannon, observed OTUs, Goods coverage, and Fisher index).*

### Co-occurrence Patterns of Soil Bacteria in Five Ecological Types

**FIGURE 7:**
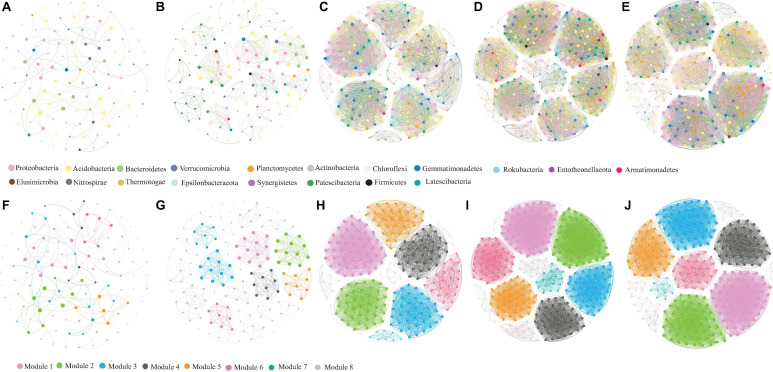
Soil bacterial co-occurrence networks in CL **(A,F)**, GL **(B,G)**, PF **(C,H)**, AF **(D,I)**, and RD **(E,J)** based on correlation analysis. The nodes in network **(A–E)** are colored according to phylum, while the nodes in network **(F–J)** are colored with respect to modularity class. The size of each node is proportional to the number of connections.

Co-occurrence network analysis revealed remarkable differences in five ecological types (Figure 7). The clustering coefficient in CL, GL, PF, AF, and RD was 0.783, 0.986, 0.996, 0.997, and 0.997, respectively, and the modularity index in CL, GL, PF, AF, and RD was 0.837, 0.908, 0.812, 0.826, and 0.817, respectively. The resulting soil microbial networks in CL, GL, PF, AF, and RD were consisted of 113, 153, 249, 344 and 318 nodes (OTUs) as well as 233, 581, 4314, 7405, and 6768 edges, respectively. The average degree or node connectivity in CL, GL, PF, AF, and RD was 4.12, 7.60, 34.65, 43.05, and 42.57, respectively. In addition, the soil microbial network in GL, PF, AF, and RD was comprised of highly connected OTUs (37.97, 17.73, 21.53, and 21.28 edges per node, respectively) structured among densely connected groups of modules (nodes), where a clustered topology was formed. In contrast, the soil microbial network in CL was comprised of lowly connected OTUs (2.06 edges per node).

All the top six bacteria in five co-occurrence networks were classified into Proteobacteria, Acidobacteria, Bacteroidetes, Verrucomicrobia, Gemmatimonadetes, Actinobacteria, Chloroflexi and Planctomycetes. Interestingly, Firmicutes dominated in the co-occurrence network of CL and Actinobacteria dominated in the co-occurrence network of GL, PF, AF and RD. Besides, *Candidatus Udaeobacter*, *Chthoniobacterales*, and *Pedosphaeraceae* were identified as the top three genera in five co-occurrence networks.

## Discussion

### Responses of Bulk Soil Physicochemical Characteristics to Ecological Types in KRD Control Area

Reforestation can increase SOC accumulation and N stock across the chronosequence (Li et al., 2012, 2021; Tong et al., 2018). In the present study, the generally increased soil TN and SOC from grass land to secondary forest land (that is, AF or PF) and natural land (that is, RD) infers that returning farmland to forests promotes the accumulation of organic materials in KRD control areas, in accordance with previous studies (Xue et al., 2017; Tong et al., 2018). The link between ecosystem restoration and SOC as well as N accumulation is driven by the translation of plant residues and root exudation to belowground C and N storage (Shao et al., 2019). Moreover, rich calcium in karst soil plays another active role in SOC stabilization (Rowley et al., 2018). In contrast, low mean SOC content with 27.13 g/kg in corn land (the mean SOC content in croplands of southwest China is 24.5 g/kg) may have been due to the combined application of farm manure and chemical fertilizer. Though the mean soil C:N ratios in China was 11.9 (Tian et al., 2010), the soil samples in our study had the mean soil C:N ratios > 25.44, which generally decreased from grass land to secondary forest land and natural land. The high C:N ratios in our study are driven by additional biotic factors (Shao et al., 2019) and tightly linked to soil, water and nutrient loss/leakage in karst regions with the karst fissures or karren characters, which have been observed by the continuing drain of NO_3_^–^ from soil into epikarst spring (Li et al., 2010; Peng et al., 2019). In addition to ecosystem restoration effects on decreased C:N ratios during reforestation chronosequence, we speculate that ectomycorrhizal fungi that can selective decompose and uptake organic N will drive N limitation of plant roots and free-living decomposers, which is consistent with slowing soil C respiration and increased soil C storage, as previous observed (Averill and Hawkes, 2016). Unlike the soil C and N, soil P is majorly provided by the weathering of the parent material (Walker and Adams, 1958). In this study, soil TP exhibited a high spatial heterogeneity and all soil samples had mean C:P ratios <38 (the mean soil C:P ratios in China was 61) (Tian et al., 2010), indicating that C:P ratios might change with environmental factors although “Redfield-like” interactions among C and P may exist in soil (Cleveland and Liptzin, 2007). Moreover, the changed C:P ratios and TP content may be due to soil P leaking dominated by the interactive effects of runoff losses, and temperature or microorganism influence on weathered soils in tropical-subtropical regions (Richardson and Simpson, 2011; Peng et al., 2019). Thus, we found that TP content almost decreased and C:P ratios almost increased across the chronosequence, suggesting high P input in cropland and low P supply leading to high C:P (Tian et al., 2010). In contrast, the land covered by *Pinus yunnanensis* forest exhibited low C:P ratios, which may be relating to high soil weathering rate caused by coniferous afforestation with soil acidification activity (Brand et al., 1986). Considering that soil C:P ratios <200 implies net mineralization (Paul, 2007) and soil C:N ratios > 25 indicates organic matter accumulation faster than decomposition (Bui and Henderson, 2013), our results inferred N and P co-limitation in the karst ecosystem.

In carbonate area, calcareous soil can reduce acidification activity caused by plants and soil disturbances, consequently the soil exhibited pH values > 6.24. While the role of plants in reusing soil magnesium and in uptaking calcium accumulated in plant leaves has been well established (Marschner, 2011), soil calcium and magnesium as the essential mineral elements for plants and microbes exhibited high spatial heterogeneity. However, the changed edaphic properties during ecosystem restoration cannot be adequately explained without soil microorganism which is not only the main engines and biomass of biogeochemical cycle in terrestrial ecosystem (Delgado-Baquerizo et al., 2018, 2019; Wagg et al., 2019). In this respect, we speculate the changed edaphic properties are driven by biotic factors, specifically bacteria, the quantitatively dominant microorganisms in soil ecosystems, that link ecosystem restoration and soil physicochemical variation (Delgado-Baquerizo et al., 2018, 2019; Shao et al., 2019; Wagg et al., 2019).

### The Impacts of Ecological Types on the Differentiation of Soil Bacterial Communities in KRD Control Area

This study and many previous studies showed that vegetation restoration influenced soil properties (Jiang et al., 2014; Li et al., 2014; Chang et al., 2018; Bai et al., 2019). Considering that microbial processes relating to element cycling in soil, the changed soil properties in turn can affect soil bacterial communities (Delgado-Baquerizo et al., 2018, 2019; Wagg et al., 2019). In the present study, the differentiation of soil bacterial communities in five ecological types indicated that returning farmland to forests might play important roles in shaping soil bacterial community diversity, as shown by ANOSIM analysis and PCoA plots. However, the similarity between corn land and *Pinus yunnanensis* forest was observed based on both ANOSIM analysis and PCoA plots, indicating that managed ecosystems have the similar beta diversity, which is a key concept about what controls diversity in ecological communities (Condit et al., 2002). That is, artificial selection illustrates an important role in shaping the whole ecosystems composed by thousands of species and millions of individuals (Swenson et al., 2000). In contrast, an enhanced dissimilarity between the natural ecosystems and the sustainable management ecosystems was observed in our study. Previous studies have shown that soil bacterial community structures can be shaped by environmental factors, such as pH (Fierer and Jackson, 2006) and calcium (Xue et al., 2017). In the present study, soil pH and E-Ca were closely correlated with soil bacterial distribution in five ecological types. The strong correlation between soil pH and bacterial distribution may have been due to each type of microorganism having an optimal pH value, and a slight change in pH favoring distinct bacterial taxa (Fierer and Jackson, 2006; Xue et al., 2017). Calcium is the important nutrient required by bacteria for growth and cell maintenance, signaling pathways regulating, gene regulation, cellular communications and responsiveness to both the intracellular and extracellular environments, bio-macromolecular structural modification, and bacterial biofilm formation (Xue et al., 2017; Wang et al., 2019). On the contrary, bacteria can induce calcium carbonate precipitation as part of their basic metabolic activities in karst soil providing sufficient calcium and carbonate ions (Dhami et al., 2013), which has been linked to sequestration of atmospheric CO_2_ (Li et al., 2015b). Therefore, the significant positive correlations between soil bacterial distribution and pH as well as calcium imply that they are the important factors for shaping soil bacterial communities. Microorganisms are integral to soil P cycle and a small component of total soil P is directly available for microbial uptake (Richardson and Simpson, 2011). In this study, soil bacterial distribution demonstrated significant positive correlations with phosphorus, which implies that the available P content acts as a determining factor in shaping soil bacterial populations. The P limitation can increase the concentrations of microbial residues and might be favorable for the stability of SOC by through affecting the soil bacterial communities (Yuan et al., 2020). This findings are supported by significant relationship between C:P ratios and soil bacterial community composition. In contrast, C:N ratios have no significant relationships with soil bacterial diversity and community composition, though the significant relationships of C:N ratio with bacterial diversity and community composition have been reported by Xue et al. (2017). This is unsurprising, however, considering that soil, the continuous spatio-temporal heterogeneity with high spatial variability, harbors vast soil microorganisms (Yuan, 1997; Harris, 2009; Jiang et al., 2014; Delgado-Baquerizo et al., 2018, 2019), where bacteria play an important role in the metabolism of one-C compounds and fungi are the key drivers of decomposition processes (Delgado-Baquerizo et al., 2018, 2019; Shao et al., 2019; Wagg et al., 2019). Though fungal dynamics in response to environmental gradients is much scarcer than for bacterial communities (Bastida et al., 2016), different bacteria to fungi ratios altered by the habitat changes induced the different contributions from microbial catabolism and/or anabolism driving soil C and N dynamics (Wagg et al., 2019; Zhong et al., 2020). Therefore, we speculate the changed allocation between plant and soil microorganisms may lead to the out-sync with carbon and nitrogen, though the contribution of fungi was not directly evaluate in our study. In addition, plant Gleason index is another important factor driving karst soil bacterial distribution. That is, plant diversity can enhance the range of food resources available for soil microbes (Van der Heijden et al., 2008; Lamb et al., 2011; Lange et al., 2014; Zuo et al., 2016). But despite all that, we could not identify pH, calcium, phosphorus, plant richness, or all four, which is the key factor(s) determining microbial community structure.

Instead of focusing on the relationship between individual edaphic factors and soil bacterial communities, latent variables of plant richness, soil properties, soil bacterial communities, and soil bacterial diversity as combinations of related factors were used in our PLS-PM, which could well interpret the inter-relationships among variables (Tenenhaus et al., 2005; Zhong et al., 2020). In the present study, it was found that plant richness had important indirect effects on soil bacterial community diversity and composition mainly through the effects on soil properties, as exhibited by Supplementary Figure 3. Recently, several studies have shown that community assembly of soil bacteria was connected to ecosystems (Delgado-Baquerizo et al., 2018, 2019; Wagg et al., 2019) and concluded that greater diversity would promote the metabolism of plant inputs in soil and hence alter the compositions and functionalities of the soil microbial community (Wagg et al., 2019). Here, our result provided evidence that soil bacterial community was mediated by ecological types through the changed soil properties.

### The Contributions of KRD Control to Soil Bacterial Communities

The interactions among the vast diversity of microbes in soil are direct or indirect, such as competition, facilitation, and inhibition (Wagg et al., 2019), which can be reveled by microbial co-occurrence analyses (Barberán et al., 2012; Ma et al., 2016; Xue et al., 2017). The modularity index > 0.4 indicates that the network has a modular structure (Xue et al., 2017). The most abundant phyla in the soil bacterial co-occurrence network showed that Firmicutes were dominating in CL and Actinobacteria were dominating in GL, PF, AF, and RD. That is, all these members of the phylum Firmicutes are well known for their facultative to strict anaerobic metabolism (Johnson et al., 2008). Extreme environmental conditions such as karst farmlands can favor Endospore-forming Firmicutes deploying a myriad of survival strategies to resist adverse conditions (Filippidou et al., 2016; Gupta et al., 2018). In contrast, Actinobacteria are known as degraders of highly recalcitrant organic materials, including substances recycling, polymer degradation, and bioactive molecule production (Barka et al., 2016). The link between ecosystem restoration and dominant Actinobacteria suggests that the decomposition, transformation and stabilization of the accumulated organic materials via input of plant residues and root exudation, and the contribution of microbial necromass are driven by this dominant bacterial functional group (Barka et al., 2016; Delgado-Baquerizo et al., 2018). Thus, it is critical that future research describes the contribution of actinobacterial species in biogeochemical cycles of organic materials. Though Acidobacteria, Bacteroidetes, Proteobacteria, Chloroflexi, Verrucomicrobia, Planctomycetes, Gemmatimonadetes were the top 7 phyla in the co-occurrence network, only Verrucomicrobia demonstrated stronger correlations with ecological types, indicating that these dominant bacteria will be critical drivers, or indicators, of key soil processes worldwide (Delgado-Baquerizo et al., 2018). Verrucomicrobia usually increase consistently with ecological succession (Bergmann et al., 2011; Jangid et al., 2011; Shen et al., 2017) and have the oligotrophic and slow growth rate life history strategy (Bergmann et al., 2011), which allows them exploit sparse resources (such as N and P co-limitation) in karst soils. The fact that three top OTUs (*Candidatus Udaeobacter*, *Chthoniobacterales*, and *Pedosphaeraceae*) demonstrating stronger correlations with ecological types were also recognized in the co-occurrence networks, indicating their important ecosystem multifunctionality involved in SOC accumulation and N stock across the chronosequence. *Candidatus Udaeobacter*, an aerobic heterotroph encoding many amino acid and vitamin transporters, which can minimize cellular architecture and sacrifice metabolic versatility for efficiency to become dominant in the soil environment, have the capacity to store surplus carbon as glycogen or starch (Brewer et al., 2016). *Chthoniobacterales* are very common in soils (Sangwan et al., 2004) and can make rapid responses to changes in the availability of substrates and oxygen in soil (Hirsch et al., 2017). *Pedosphaeraceae* has been identified as an important species in soil biogeochemical cycle, yet a better understanding of their roles in soil bacterial co-occurrence networks is still needed in the future (Zhang et al., 2019). However, a recent study revealed that *Bryobacter*, GR-WP33-30, and *Rhizomicrobium* were the keystone karst bacteria in Fuyuan County, Yunnan Province (Xue et al., 2017). This is unsurprising, however, considering that only a small fraction of soil bacteria is typically shared between any pair of unique soil samples (Delgado-Baquerizo et al., 2018). Therefore, the three top OTUs in our study will be critical drivers, or indicators, of key soil biogeochemical processes. Though rare soil bacteria are also the drivers of key functions in terrestrial ecosystems, which may be due to effects that are disproportionately large given their abundance or via the provision of insurance effects (Jousset et al., 2017), their roles in biogeochemical cycles is far less clear (Liang et al., 2020). Thus, it is critical that future research describes the contribution of rare soil bacteria driving ecosystem multifunctionality.

Soil microbial network complexity varies with their habitat conditions (Ma et al., 2016; Banerjee et al., 2019; Xue et al., 2020). For instance, soil microbial network complexity in late successional fields was higher than that in early successional fields (Morriën et al., 2017) and agricultural intensification can reduce soil microbial network complexity (Banerjee et al., 2019). Besides, soil microbial network complexity indicates the stage of ecosystem stability and multifunctionality (Wagg et al., 2019). In the present study, soil microbial network complexity increased from corn land to grass land, forest and natural land, indicating that the lower network complexity of soil bacteria in corn land need additional management where nutrient-use efficiency is low and degradation of soil quality is serious, which accords with a recent study on KRD process in southwest China (Jiang et al., 2014). That is, disturbances such as cultivation and fertilization might eliminate certain functional soil bacteria (Neher, 1999; Huang et al., 2019). In contrast, soil microbial network complexity is much higher in natural land, though attainment of steady state equilibrium in natural ecosystems is rare (Neher, 1999). Based on global meta-analyses with more than 200 reforestation samples, it was found that ecosystem restoration, rather than the geographical distribution, is the dominant factor influencing the complexities of the soil microbiome (Shao et al., 2019). Compared to natural secondary forests (*Alnus ferdinandi-coburgii* Schneid), the artificial secondary *Pinus yunnanensis* forests had the lower soil bacteria network complexity, which might be relating to the outbreaks of pine moth in Yunnan province, China due to the loss of certain functional soil microbe which can produce secondary metabolites in suppressing pathogens and against harmful insert (Schmidt et al., 2019). Therefore, the rich soil microbiota tends to suppress the severity of the attack by many soilborne plant pathogens (Garbeva et al., 2004). Moreover, the clustering coefficient values of soil bacterial co-occurrence network in AF similar to RD was higher than that in PF, which demonstrates the similar microbe network complexity in natural secondary forests and natural ecosystem (Xue et al., 2017). It is possible that artificial re-vegetation promotes the ecological restoration and environment improvement, however, changes in belowground ecosystem lag behind the corresponding changes in aboveground ecosystem (Delgado-Baquerizo et al., 2018). By referring to the co-occurrence patterns of belowground ecosystem in future studies at a broader spatial-temporal scale in karst area, our result paves the way for predictive understanding the belowground diversity during ecosystem restoration, which in turn can help us to amend the strategy and orientation of ecological restoration.

## Conclusion

In the present study, we revealed the differentiation of soil bacterial communities in five ecological types in KRD control area. Our work suggested that plant richness was the key factor determining soil bacteria community, and soil microbial network complexity and stability. The key taxa, *Candidatus Udaeobacter*, *Chthoniobacterales*, and *Pedosphaeraceae*, which are classified into Verrucomicrobia, played the key roles in karst soil ecosystems. Finally, we demonstrated that natural secondary forests could promote the stability of soil ecosystems compared with the artificial secondary forests. That is, natural recovery is the suitable way for restoration and rehabilitation of degraded ecosystems. Thus, our study provide the methodology to appraise the interactions among soil-plant ecological network, though there is still a lot of work left to be done in the future.

## Data Availability Statement

The achieved raw sequence reads were submitted to the NCBI Sequence Read Archive under the accession numbers PRJNA471031 and PRJNA471162.

## Author Contributions

HY completed the soil sample collection. AS completed the data analysis and DNA extraction. QL and WM prepared the manuscript draft. QL designed the experiment, analyzed the data, and wrote the manuscript. WM helped with polishing the text and improving the structure and logic of the manuscript. All authors contributed to the article and approved the submitted version.

## Conflict of Interest

The authors declare that the research was conducted in the absence of any commercial or financial relationships that could be construed as a potential conflict of interest.

## Footnotes

1 http://www.r-project.org

## Supplementary Material

Supplementary Figure 1 The two relations of ANOSIM analysis in five ecological types based on OTU abundance. Ordinate–the rank of the distance between samples; Abscissa–Between is the result between every two ecological types, and the other two are the results within their groups, respectively.

Supplementary Figure 2 Changes of *Candidatus Udaeobacter*, *Chthoniobacterales*, and *Pedosphaeraceae* with plant Gleason index.

Supplementary Figure 3 VPA shows the effects of plant richness and soil properties on soil bacterial community compositions **(A)** and alpha diversities **(B)**.

Supplementary Table 1 The bacterial taxonomy of the soil samples.
