# Supplementary figures and images for: Impact of Rocky Desertification Control on Soil Bacterial Community in Karst Graben Basin, Southwestern China
Source: Front Microbiol. 2021 Mar 10;12:636405. doi: 10.3389/fmicb.2021.636405 (PMC8006366; doi:10.3389/fmicb.2021.636405)

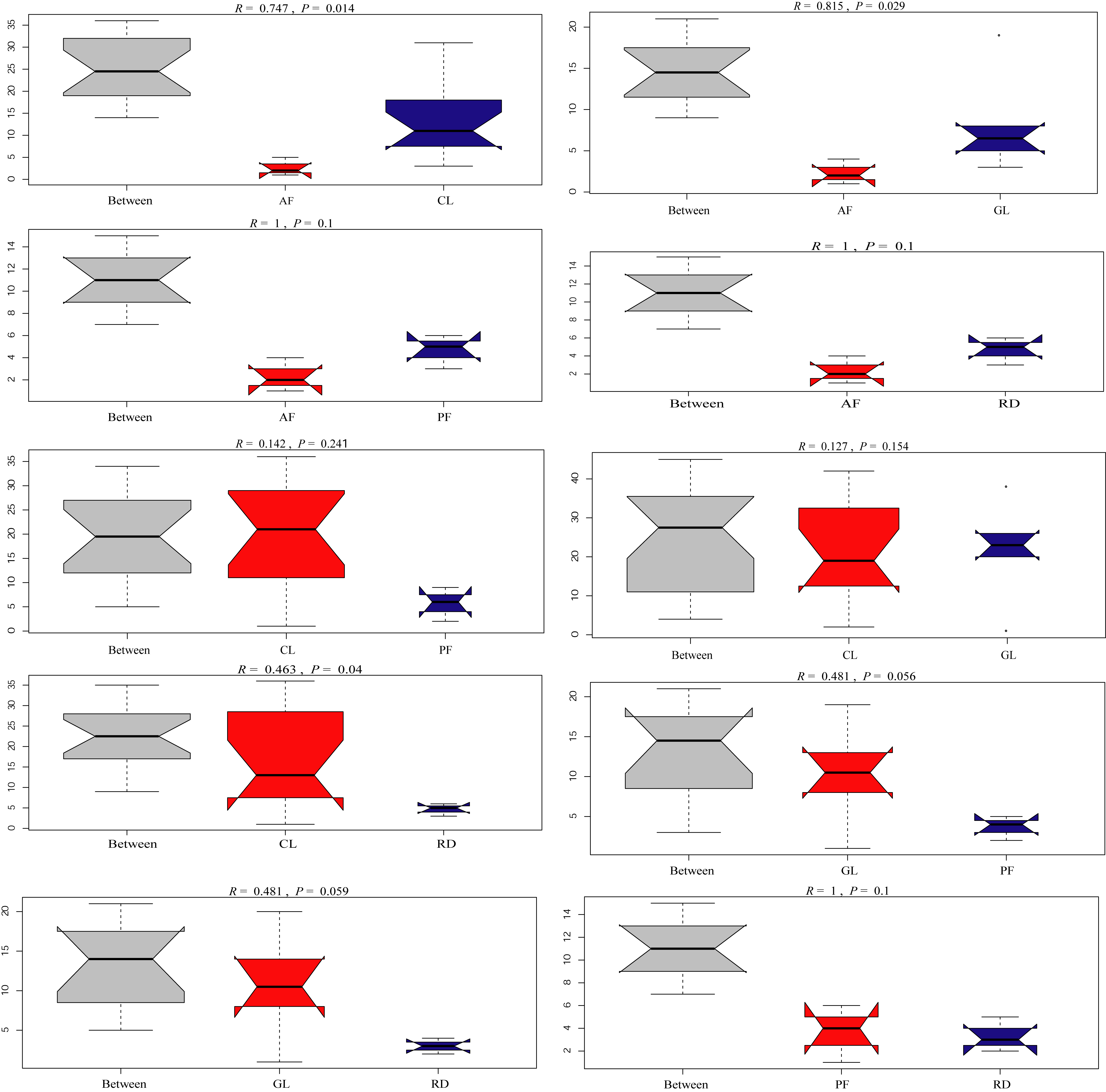

Supplement: Supplementary Figure 1 — The two relations of ANOSIM analysis in five ecological types based on OTU abundance. Ordinate–the rank of the distance between samples; Abscissa–Between is the result between every two ecological types, and the other two are the results within their groups, respectively. [file Image_1.PNG]

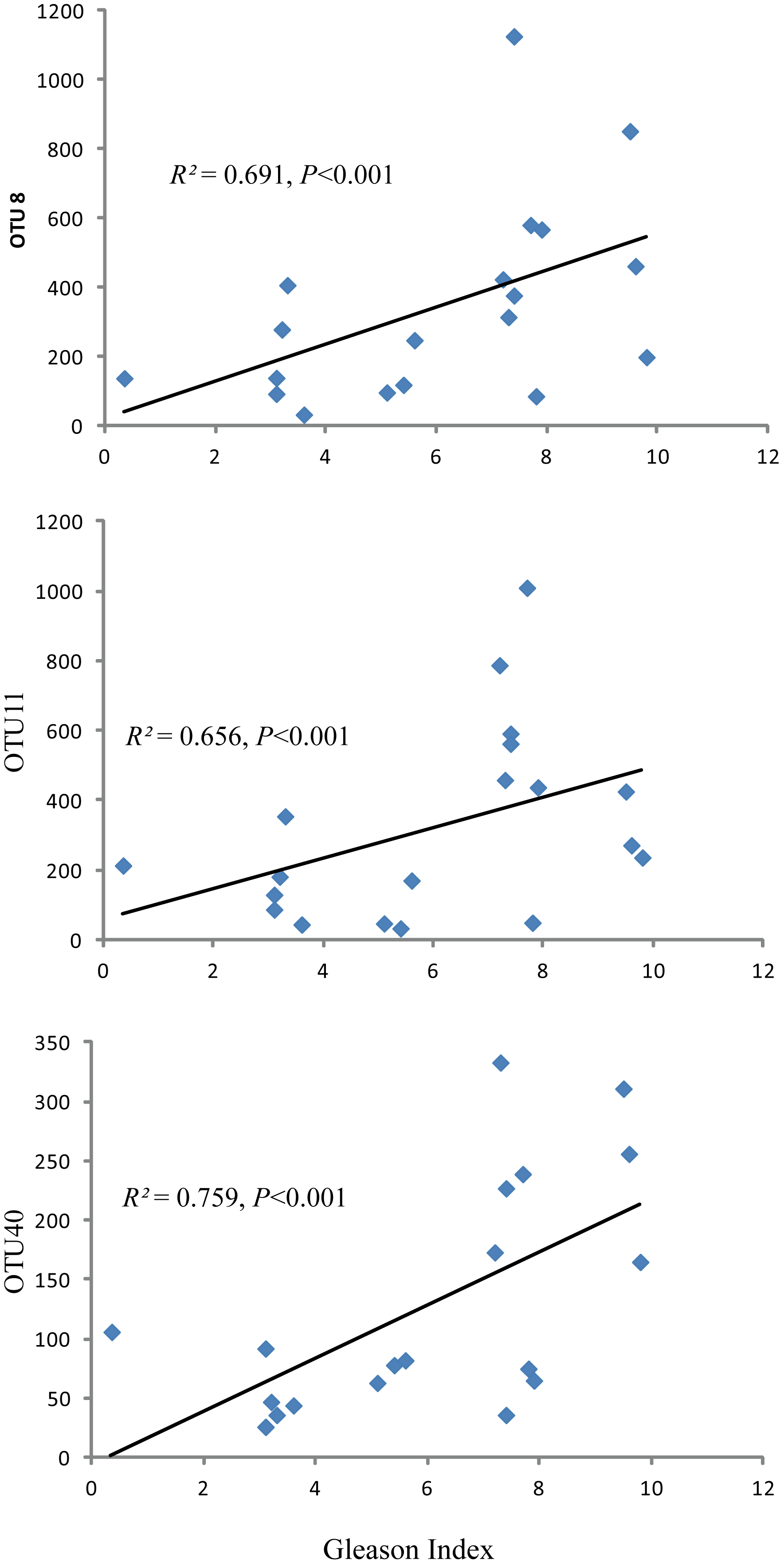

Supplement: Supplementary Figure 2 — Changes of Candidatus Udaeobacter, Chthoniobacterales, and Pedosphaeraceae with plant Gleason index. [file Image_2.PNG]

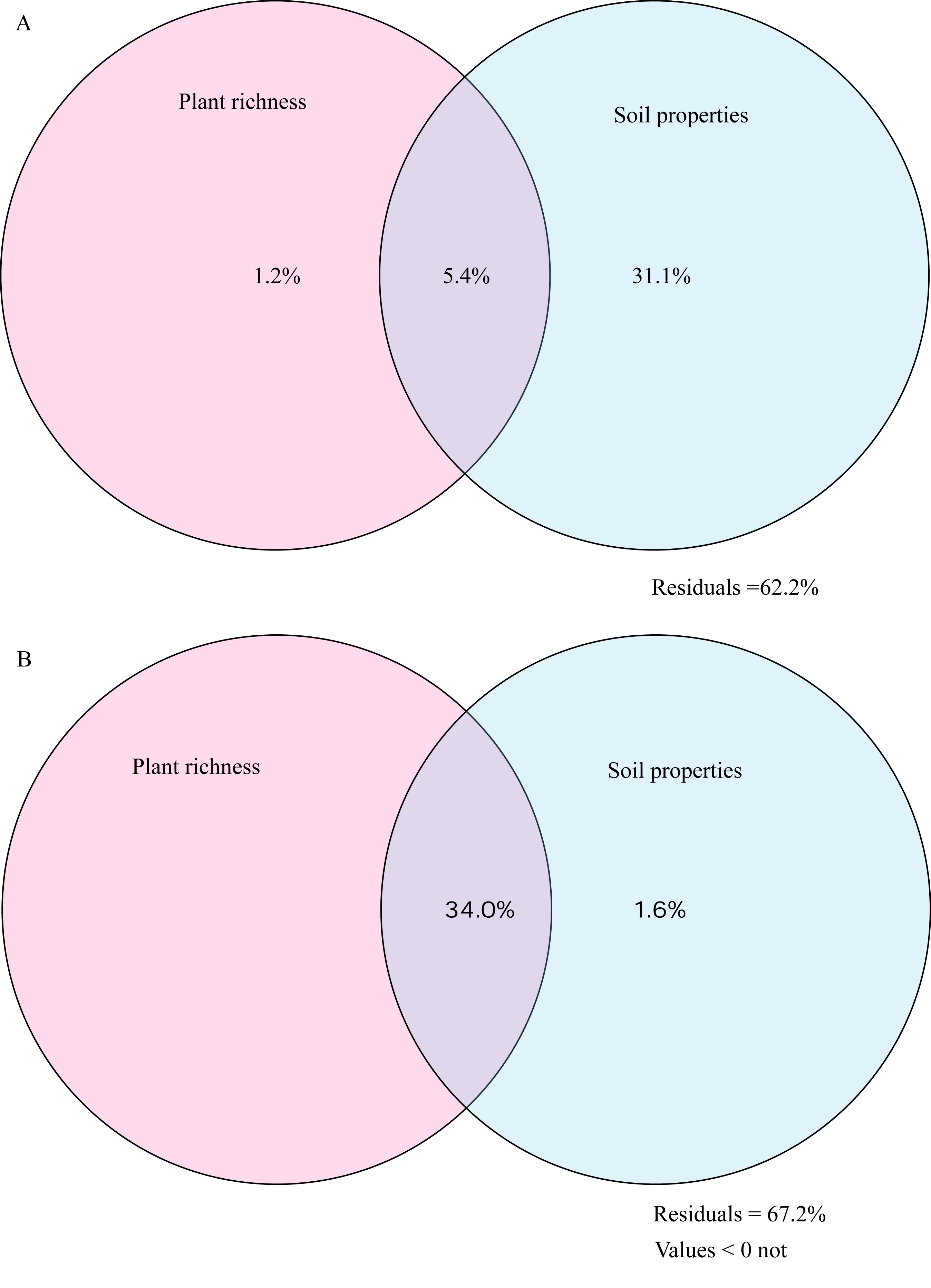

Supplement: Supplementary Figure 3 — VPA shows the effects of plant richness and soil properties on soil bacterial community compositions (A) and alpha diversities (B). [file Image_3.JPEG]
